# Cultural and Religious Variation in Attitudes to Young People Consenting to Health Interventions

**DOI:** 10.1007/s10943-018-0686-z

**Published:** 2018-08-21

**Authors:** Glòria Durà-Vilà, Matthew Hodes

**Affiliations:** 1 University College London, London, UK; 2 Imperial College London, London, UK

**Keywords:** UK, Spain, Culture, Consent, Ethics, Psychotherapy, Young people

## Abstract

There is a limited amount of empirical data available regarding the cultural and religious variation in perceptions about the age when young people should be regarded as competent to make decisions in health settings. A public survey of 400 adults from diverse religious and ethnic backgrounds was conducted in the UK and Spain. Attitudes were assessed using case vignettes. It was found that high religious practice was associated with recommending a higher age of consent for medical interventions. White British adults were more likely than Spanish adults to agree that younger adolescents should be allowed to consent to medical interventions. The study suggests that there is social, cultural and religious variation in adults’ attitudes regarding the age when youngsters should consent to health interventions.

## Introduction

It is an established principle of good clinical practice that the competent individual consents to treatment offered by medical practitioners and allied professionals. This is anchored to the key principle from medical ethics that the individual has autonomy and is entitled to make the decision about treatment free from coercion by health professionals or family and friends (Beauchamp and Childress 2001). However there are two areas in which these established principles are not only uncertain and controversial, but may actually be challenged and rejected. Firstly, these concern the culturally shaped understanding of autonomy, and the extent to which this is recognized, and expressed, including in health settings. Secondly, it is recognized that the notion of autonomy needs to be adjusted with regard to children, for whom parents and carers may consent. However, the age at which young people may consent to treatment is contested. One aspect of this is the cultural variation in parents’ views about children’s and young people’s ability and right to consent. This paper is concerned with the intersection between these two areas: the cultural construction of autonomy and right to consent to treatment; and the cultural variation in attitudes to the age at which youngsters should be entitled to consent. Issues of lack of competence to consent because of physical illness, or the use of coercion within psychiatric settings for mental health disorders, are outside the remit of this paper.

The European and North American understanding of the individual assumes the person has a sense of self which is the basis for rational action and expression of affect. The origin of this is culturally and historically shaped and can be seen in the individualism originating in the philosophy of Locke and J. S. Mill (Lukes 2006). The individual is expected to shape key events over his/her life which include negotiating or contributing to arrangements for marriage, as well as participation in economic activity. This tradition is reflected in the importance of the individual, and autonomy, in medical decision-making and medical ethics (Beauchamp and Childress 2001). Within psychotherapy, the individual has a sense of self which can be meaningfully communicated as subjective experience (Kirmayer 2007).

This individualist notion of the self can be contrasted with the collectivist idea in which the self is only meaningful in the context of other individuals. The individual may be linked to deceased relatives through vertical ties that have a religious or spiritual basis and the continuity of the vertical tie links parents to offspring. Gender differences may operate, so that the female individual and self is subordinated to the male in the public domain (the worlds of work and politics), although there may be more equality or even dominance in the domestic sphere. Cultural variation in views of the self also needs to be taken into account (Markus and Kitayama 1991). Many societies in Africa, South East Asia and China have a collectivist orientation (Dumont 1980; Marsella et al. 1985). A good example comes from China, where the Confucian influence considers the individual to be embedded in vertical ties, parent to offspring. Males and females are complementary (the spiritual notion of opposites, yin and yang underlying this). The family is the meaningful social unit and within this framework the sick individual does not traditionally make decisions; instead they may be made by relatives, with a view to maintaining overall harmony within the family and with their religious-cosmological beliefs (Yeo and Hikuyeda 2000). Within this framework the troubled individual’s psychological distress may be treated with family therapy, which includes the functioning social unit, the family, as the site for intervention (Lee and Mock 2005).

Across cultures, there are significant variations in parenting style. Nevertheless parenting in collectivistic societies may be associated with the expectation of stronger family orientation, reflected in living arrangements (e.g. leaving home occurs at the time of marriage when a new family starts) and in pooling of income and financial resources and labour (e.g. working in the family business). Consistent with this is a lesser importance given to children’s rights, and furthermore we also see in these societies that decision-making in clinical settings is traditionally a family affair. Adolescents from different cultural backgrounds will learn the prevailing views of their communities and have quite different attitudes with respect to the importance of autonomy (Fuligni 1998).

Parenting styles are also influenced by the parents’ religion (e.g. authoritarian parenting/educational style has been linked to Muslim parents/schools) (Dwairy 2006).^1^ Parental religious fundamentalism and right-wing authoritarianism have been linked to the promotion of less child autonomy and positively related to a stronger emphasis on their children’s obedience (Danso et al. 1997). Conservative Protestants, and to a lesser extent Catholics, place more value on obedience in children than other Americans (Ellison and Sherkat 1993).

Across societies there is an awareness that very young children cannot safely act on their autonomy and so consent to medical treatment. Within Europe and North America there has been decades of debate about children’s rights and the extent to which they can act in their best interests and give consent. Some of this debate is informed by developmental psychology. It is recognized that greater cognitive maturity would enable an older child to make medical decisions in their best interests (Fundudis 2003). Early adolescents (i.e. 14 years and older) who have normal intellectual development may be regarded as being competent (Fundudis 2003). However recent research into brain maturation during adolescence, including the frontal cortex (Giedd et al. 1999), has suggested that greater inhibitory control and capacity to reflect on the long-term harm and benefit of interventions should push the age of consent upwards (Partridge 2010).

The United Nation Conventions on the Rights of the Child (United Nations 1989/1990) has attempted to balance the rights of the child, responsibilities of the carers and parents, and responsibility of the state. The convention states that decisions should be made according to the best interests of the child. However in many situations it is unclear how the best interests of the child will be decided. The three above countervailing perspectives may diverge. In global perspective, a central aspect of this divergence is the cultural construction of responsibility, and the way in which child rights should be recognized and expressed (Iltis 2010).

In culturally diverse societies these issues make clinical practice fraught with difficulty. Working with adolescents is likely to generate difficulties, especially in those cases where adolescents disagree with their parents regarding treatment, or when there may appear to be conflicting issues of confidentiality such as the case of adolescents asking their doctors to prescribe contraceptives and to keep it from their parents (Didcock 2007; Harrison et al. 1997).

There is a limited amount of empirical data in the area of competence and consent in children and adolescents (Tan and Jones 2001). Most of the studies focus on the investigation of children’s and adolescents’ capacity to make decisions for themselves rather than looking at adults’ beliefs and attitudes on this matter. This gap is of particular importance as research has shown the significant effect of parental influence on adolescents’ decision-making regarding their treatment (Hinds et al. 2001; Scherer and Reppucci 1988). There appear to be little or no cross-cultural investigations of these issues. This study responds to the need for more cross-cultural and social investigation into adults’ attitudes regarding adolescents’ ability with regard to medical decision-making. On the basis of the previous studies mentioned above, the following hypotheses were developed:

1. There will be variation between cultural groups in the ages when the young people are permitted to make medical and health decisions. Thus, participants from more individualist societies (e.g. North Europe) will choose lower ages at which the youngsters will be regarded as able to make decisions than those from more socio-centric societies (e.g. South Asia) as well as having higher rates for allowing the young person to consent.^2^
2. Participants with more frequent religious attendance will give higher ages at which the young people are regarded as able to make medical decisions as well as having lower rates for allowing the young person to consent.
3. Those with a higher level of education will give lower ages at which the youngsters are thought to be able to make medical decisions and will more often allow them to make them.

## Methods

### Setting and Participants

A population survey was undertaken in eight adult educational centres, four in the region of Valencia^3^ (Spain) and four in London (United Kingdom). All eight centres approached agreed to participate. The Spanish centres were associated with or run by Catholic organisations: one was a Catholic theological college which had ten satellite centres in neighbouring villages, and the other three (one in the city of Valencia and two in nearby towns) offered courses to immigrants, the majority of whom were Hispanic Americans. Regarding the four centres located in London: one was a College, part of the University of London; two were local authority adult education centres accommodated within town halls; and another one was linked to a Mosque.

Teachers and administrative personnel distributed questionnaires to be self-completed among the adults attending these centres. 425 questionnaires were handed out and 400 were returned (94.1% were returned); however, although the majority of participants provided answers to most of the information requested, some left some gaps in the questionnaires. Thus, when reporting the findings the total number of participants answering a particular question has been detailed.

The inclusion criteria included having at least intermediate ability to read in Spanish or English. The teachers and administrative personnel ensured that this was the case for the participants before handing out the questionnaires. Ethical approval to undertake the study was granted by Imperial College London Research Ethics Committee. All participants gave informed consent.

### Measures

Our self-designed questionnaire included questions about socio-demographic information and questions about religious affiliation and frequency of attendance at a place of worship. Ethnicity was recorded as officially coded in Spain and UK following the standard terminology used in each country and site of religious attendance was modified according to religion (e.g. Mosque for Muslims or Church for Christians). The questionnaires were in Spanish (for the Spanish centres) and English (for the centres in London). Five hypothetical case vignettes followed, all of them portraying a young person (no age specified) confronting a medical, psychiatric or psychological intervention—which was recommended by professionals—in which there was a disagreement between the young person, wanting to receive the treatment (except for the case of a terminally ill patient who is refusing treatment), and their parents opposing it. These clinical scenarios were derived from clinical cases encountered by the authors. In each scenario the young person was portrayed in the best possible light (e.g. ‘responsible’, ‘sensible’) to facilitate the participants’ decision with regard to the minimum age required. The scenarios involved the following treatments: (1) psychotherapy sessions; (2) hospital psychiatric admission; (3) the contraceptive pill; (4) minor surgical intervention; and (5) life-sustaining treatment for a terminally ill patient. There were additional scenarios on non-health-related aspects of autonomy which are reported elsewhere (Durà-Vilà and Hodes 2017).

After each vignette, participants were asked if the young person should be permitted to give consent to receive the treatment (or refuse it for the terminally ill case) (‘yes’ or ‘no’) and, if answering ‘yes’, to write down the earliest age in years at which he/she should be allowed to have/refuse the treatment. They were finally asked, if they answered ‘no’, to choose from a number of possible options why they thought the young person should not be allowed to make the decision to receive/refuse the treatment (multiple answers were allowed).

The options were developed following a pilot questionnaire which was tested with a total of 30 volunteers in Valencia and in London (these responses were not included in the results). There were two options available for all the scenarios: ‘his/her parents should decide—they know what is best for him/her’, and ‘he/she is too young/immature to decide this’. Another option was added for the vignette portraying a terminally ill patient (‘life should be prolonged’) and two more options for the scenario where a girl seeks a prescription for the contraceptive pill (‘I don’t agree with sexual relationships outside marriage’ and ‘I don’t agree with the use of contraceptives’).

### Analysis

Pearson Chi-square tests with Yates’s correction with 95% confidence intervals (Fisher’s exact tests when required by the size of the cells) and one-way independent ANOVA (Welch’s *F* correction was used when Levene’s test was significant) were used.^4^ Finally, hierarchical logistic regression was carried out to investigate which variables might predict granting permission to the young person to make decisions about their treatments. They were identified on the basis of the strength of the association with the specified outcome variable. Only those variables which were found to be statistically associated with granting permission or which had theoretical reasons to support the association were entered in the regression (Field 2009). All analyses were conducted using the Statistical Package for Social Sciences (SPSS 17.0, 2003) for Windows, and a 95% (*P* < 0.05) statistical significance level was applied.

## Results

### Sample Characteristics

Regarding ethnicity, of the total 400 participants, half of them were Spanish (200/400, 50.0%), the rest were White British (81/400, 20.3%), Hispanic Americans (66/400, 16.5%) and South Asians (53/400, 13.2%) (South Asians included: Bangladeshi, Indian and Pakistani). All the Hispanic American responses were collected at the Spanish sites, and all the South Asians were collected in the UK. The age range was 18 to 85 years with a mean age of 45.4 years (SD = 16.9) [18–30 years (104/396, 26.3%); 31–45 years (95/396, 24.0%); 46–65 years (134/396, 33.8%), over 65 years (63/396, 15.9%)]. The majority of the sample were women (276/399, 69.2%), most participants were married or cohabiting (230/399, 57.6%) and most had children (206/388, 53.1%).

The majority of participants were Christian (317/400, 79.2%) (74.1% were Roman Catholics, 4.7% were Church of England and the remaining 21.2% had another Christian denomination (Methodist, Baptist, Evangelical and Orthodox) or did not specify one) and the rest were Muslim (48/400, 12.0%) or did not have a religious affiliation (35/400, 8.8%). Most of them attended the place of worship frequently (daily or weekly, 267/394, 67.8%); infrequent religious attendance (monthly or less than monthly) was reported in 19/394 (4.8%); and 34/394(8.6%) never attended formal worship or said they did not follow a religion.

Nearly half had a university degree (197/397, 49.6%); also almost half were unemployed (197/398, 49.5%), including pensioners, students and housewives. Regarding their living arrangements: most participants owned their homes (270/390, 69.2%); rented accommodation accounted for 98/390 (25.1%); and local authority or homeless numbered 22/390 (5.7%) (all of those who described themselves as homeless were staying with family or friends). In terms of their legal status, the majority were Spanish, British, European Union nationals or had leave to remain (362/393, 92.1%) with some admitting not to have leave to remain (31/393, 7.9%).^5^

### Total Responses

The prescription of the contraceptive pill reached the highest level of refusing permission with almost half of the participants not allowing the young person to receive this prescription, followed by over a quarter not allowing the patient to refuse further treatment in the terminally ill scenario, and by the psychiatric hospital admission and the operation vignettes which had similar responses (approximately a sixth of participants did not grant permission). The psychotherapy vignette was the one that had the lowest percentage of refusing permission: less than 10% of the participants would not have let the young person attend the psychotherapy sessions (Table 1).

**Table 1:** Responses to giving permission to the boy/girl of the vignette to make a decision regarding their medical treatment

Responses to vignettes	Psychotherapy (*n* = 387)	Psychiatric hospital admission (*n* = 396)	Contraceptives (*n* = 391)	Operation (*n* = 396)	Refusal of life-sustaining treatment (*n* = 385)
Permission given	349 (90.2%)	330 (83.3%)	217 (55.5%)	342 (86.4%)	284 (73.8%)
Earliest age in years (mean, SD, range)	13.34, 3.11, 5–21	15.02, 2.80, 5–21	16.36, 1.78, 12–21	14.22, 3.80, 5–21	15.71, 3.18, 6–21
Permission refused	38 (9.8%)	66 (16.7%)	174 (44.5%)	54 (13.6%)	101 (26.2%)
‘Parents should decide/know best’	19 (4.9%)	35 (8.8%)	21 (5.4%)	26 (6.6%)	22 (5.7%)
‘Too young/immature to decide’	7 (1.8%)	12 (3.0%)	36 (9.2%)	12 (3.0%)	22 (5.7%)
‘Life should be prolonged’	N/A	N/A	N/A	N/A	49 (12.7%)
‘I don’t agree with sexual relationships outside marriage’	N/A	N/A	105 (26.9%)	N/A	N/A
‘I don’t agree with contraceptives’	N/A	N/A	60 (15.4%)	N/A	N/A

N/A = non-applicable

### Ethnicity

Extremely statistically significant differences (*P* < 0.001) were found among ethnic groups regarding the mean age at which the young person should be allowed to make the decision in all five vignettes. We suggested in our first hypothesis that adults from more individualist societies will choose lower ages than participants from more socio-centric societies. This was clearly confirmed in all the scenarios with the White British participants having the lowest mean ages of granting permission than the rest of the ethnic groups for all vignettes, ranging from 12.08 to 15.31 years old (Table 2).

**Table 2:** Earliest age (mean age) at which the young person should be allowed to make a decision regarding their medical treatment [One-way independent ANOVA (*F*-ratio (*df*M, *df*R), *r*, *P* value)]

Variables	Psychotherapy (*n* = 387) *F*(*dfM*, dfR), *r*, *P*	Hospital admission (*n* = 396) *F*(*dfM*, dfR), *r*, *P*	Contraceptives (*n* = 391) *F*(*dfM*, *dfR*), *r*, *P*	Operation (*n* = 396) *F*(*dfM*, *dfR*), *r*, *P*	Refusal life-sustaining treatment (*n* = 385) *F*(*dfM*, *dfR*), *r*, *P*
Ethnicity^a^	*F*(3,296) = 6.16, 0.24*** Mean age, SD, range	*F*^c^(3,107.91) = 24.37, 0.47 Mean age, SD, range	*F*(3,203) = 18.79, 0.47*** Mean age, SD, range	*F*(3,303) = 18.95, 0.39*** Mean age, SD, range	*F*(3,242) = 25.787, 0.49*** Mean age, SD, range
Spanish	13.46, 3.28, 5–21	16.08, 2.33, 9–21	17.45, 1.73, 12–21	15.12, 3.13, 5–21	16.85, 2.64, 7–21
White British	12.14, 2.50, 6–16	13.05, 2.92, 5–21	15.31, 1.37, 12–18	12.08, 2.81, 5–18	13.43, 3.06, 6–21
Central/South American	14.27, 3.27, 6–18	16.31, 2.34, 10–21	16.52, 1.99, 12–21	15.37, 2.69, 6–20	17.08, 2.53, 10–21
South Asian	14.03, 2.73, 6–21	14.39, 2.87, 9–21	16.31, 2.15, 12–21	13.68, 3.68, 6–21	16.41, 2.58, 10–21
Religious practice^b^	F(3,292) = 2.85, 0.17* Mean age, SD, range	F^c^(3,47.02) = 5.85, 0.26** Mean age, SD, range	F(3,201) = 11.24, 0.38*** Mean age, SD, range	F(3,299) = 3.09, 0.17* Mean age, SD, range	F(3,239) = 3.78, 0.21* Mean age, SD, range
Frequent	13.69, 3.09, 5–21	15.61, 2.62, 9–21	17.04, 1.73, 13–21	14.62, 3.33, 5–21	16.34, 2.91, 7–21
Infrequent	12.77, 3.02, 6–21	14.72, 2.88, 7–21	16.04, 1.98, 12–21	13.46, 3.46, 5–21	15.28, 3.44, 8–21
Never	12.62, 3.97, 6–18	14.33, 3.70, 8–18	17.17, 1.19, 15–18	14.71, 2.64, 10–18	16.23, 3.65, 6–21
No religion	12.32, 2.87, 6–18	13.28, 3.28, 5–18	15.10, 1.73, 12–21	13.23, 2.94, 6–18	14.46, 3.07, 7–21
Education	F(2,295) = 1.92, 0.11, ns Mean age, SD, range	F(2,294) = 7.49, 0.22** Mean age, SD, range	F(2,204) = 5.37, 0.22** Mean age, SD, range	F(2,303) = 4.34, 0.17* Mean age, SD, range	F(2,242) = 2.46, 0.14, ns Mean age, SD, range
School (primary/secondary)	13.79, 3.21, 5–21	15.77, 2.76, 5–21	17.23, 2.03, 13–21	15.08, 3.25, 5–20	16.12, 2.85, 9–21
Further education	13.62, 3.31, 6–18	15.81, 2.41, 10–20	16.54, 1.89, 12–21	14.36, 3.09, 6–21	16.54, 3.38, 6–21
University	13.02, 2.96, 6–21	14.53, 2.99, 7–21	16.14, 1.83, 12–21	13.79, 3.36, 5–21	15.48, 3.14, 7–21

*df*M, degrees of freedom for the effect of the model; *df*R, degrees of freedom for the residuals of the model; *r*, effect side; *ns* no statistically significant

^a^Ethnicity as officially coded in Spain and UK. South Asians included: Bangladeshi, Indian and Pakistani

^b^Frequent religious practice: daily and weekly attendance to a place of worship. Infrequent religious practice: monthly and less than monthly attendance to a place of worship. Place of worship was modified according to religious context

^c^As the ANOVA homogeneity of variance of assumption was broken (Levene’s test was significant), Welch’s *F* correction was used

*P < 0.05; **P <0.01; ***P < 0.001

Similarly, statistically significant ethnic variation was also found in allowing the young person to make the decision regarding their treatments for all vignettes except the psychotherapy one, with White British being the ones who more frequently granted permission (over 87%) (Table 3). Moreover, binary logistic regression models revealed that ethnicity was a statistically significant predictor for the psychiatric hospital admission and refusal of life-sustaining treatment scenarios (Tables 3, 4, 5).

**Table 3:** Variables associated with giving permission/allowing the boy/girl of the vignette to make a decision regarding their medical treatment [Chi-Sq. (value, *df*) or Fisher Ex. (value) and *P* value]

He/she should be allowed to make a decision regarding	Psychotherapy χ^2^/*F* (value, *df*), *P*	Hospital admission χ^2^/*F* (value, *df*), *P*	Contraceptives χ^2^/*F* (value, *df*), *P*	Operation χ^2^/*F* (value, *df*), *P*	Refusal life-sustaining treatment χ^2^/*F* (value, *df*), *P*
Ethnicity^a^	3.57, ns	21.95, 3***	62.89, 3***	11.17, 3*	21.73, 3***
Spanish	172/196(87.8%)	154/199(77.4%)	83/194(42.8%)	166/198(83.8%)	140/195(71.8%)
White British	77/81(95.1%)	79/81(97.5%)	71/81(87.7%)	79/81(97.5%)	74/80(92.5%)
Central/South American	56/63(88.9%)	49/64(76.6%)	46/65(70.8%)	52/64(81.3%)	43/64(67.2%)
South Asian^a^	43/47(91.5%)	48/52(92.3%)	17/51(33.3%)	45/53(84.9%)	27/46(58.7%)
Religious practice	14.62**	15.76**	75.55***	8.410*	18.43**
Frequent^b^	226/261(86.6%)	208/264(78.8%)	109/261(41.8%)	219/264(83.0%)	175/259(67.6%)
Infrequent^c^	69/70(98.6%)	66/73(90.4%)	59/71(83.1%)	67/73(91.8%)	58/69(84.1%)
Never	15/17(88.2%)	19/19(100.0%)	14/19(73.7%)	18/19(94.7%)	17/18(94.4%)
No religion	33/33(100.0%)	33/34(97.1%)	33/34(97.1%)	33/34(97.1%)	30/33(90.9%)
Type of religion	0.11, 1, ns	3.30, 1, ns	13.55, 1***	0.73, 1, ns	7.11, 1*
Christian	276/310(89.0%)	253/314(80.6%)	171/310(55.16%)	269/313(85.9%)	230/309(74.4%)
Muslim	39/43(90.7%)	43/47(91.5%)	12/46(26.09%)	39/48(81.3%)	23/42(54.8%)
Education	4.26, 2, ns	4.54, 2, ns	16.14, 2***	1.61, 2, ns	0.64, 2, ns
School (primary/secondary)	91/106(85.8%)	82/107(76.6%)	43/106(40.6%)	93/108(86.1%)	74/104(71.2%)
Further education	79/89(88.8%)	77/90(85.6%)	48/87(55.2%)	75/90(83.3%)	67/88(76.1%)
University	176/189(93.1%)	168/196(85.7%)	126/195(64.6%)	153/195(88.7%)	141/190(74.2%)
Employment	6.68, 1*	0.02, 1, ns	12.04, 1**	1.67, 1, ns	1.43, 1, ns
Employed^e^	182/194(93.8%)	166/200(83.0%)	128/199(64.3%)	177/200(88.5%)	148/194(76.3%)
Unemployed^f^	164/191(85.9%)	162/194(83.5%)	89/190(46.8%)	163/194(84.0%)	134/189(70.9%)
Gender	0.23, 1, ns	7.11, 1**	0.38, 1, ns	0.23, 1, ns	5.07, 1*
Male	106/119(89.1%)	111/122(91.0%)	69/119(58.0%)	108/123(87.8%)	78/118(66.1%)
Female	242/267(90.6%)	219/273(80.2%)	148/271(54.6%)	234/272(86.0%)	205/266(77.1%)
Age	5.75, 3, ns	8.25, 3*	14.54, 3**	14.26, 3**	13.29, 3**
18–30 years	94/100(94.0%)	85/101(84.2%)	63/101(62.4%)	80/102(78.4%)	68/100(68.0%)
31–45 years	83/88(94.3%)	79/94(84.0%)	63/93(67.7%)	84/94(89.4%)	70/89(78.7%)
46–65 years	115/133(86.5%)	103/133(77.4%)	60/132(45.5%)	112/132(84.8%)	89/130(68.5%)
Over 65 years	54/61(88.5%)	59/63(93.7%)	29/61(47.5%)	62/63(98.4%)	55/61(90.2%)

χ^2^, Chi-Square; *F*, Fisher Exact test; *df*, degree of freedom; *ns*, no statistically significant

*P < 0.05; **P <0.01; ***P < 0.001

^a^Ethnicity as officially coded in Spain and UK. South Asians included: Bangladeshi, Indian and Pakistani

^b^Frequent religious practice: daily and weekly attendance to a place of worship (modified according to religious context)

^c^Infrequent religious practice: monthly and less than monthly attendance to a place of worship (modified according to religious context)

^d^Christian denominations specified by participants: Catholic, Methodist, Church of England, Baptist, Evangelical and Orthodox

^e^Employed included full-time, part-time and self-employed

^f^Unemployed included retired, students and housewives

**Table 4:** Binary logistic regression analysis for variables predicting giving permission to the boy/girl of the vignette (psychotherapy, psychiatric hospital admission and operation) to make a decision regarding their medical treatment [B(SE), OR, 95% CI of OR, *P* value]

Psychotherapy					Psychiatric hospital admission					Operation				
	B(SE)	OR	95% CI	*P*		B(SE)	OR	95% CI	*P*		B(SE)	OR	95% CI	*P*
Step 1					Step 1					Step 1				
Constant	4.88(1.16)				Constant	3.17(0.44)				Constant	0.87(0.34)			
Religious practice	− 0.98(0.40)	0.37	0.17–0.82	*	Ethnicity	− 0.40(0.10)	0.67	0.55–0.81	***	Age	0.44(0.15)	1.55	1.16–2.09	**
Step 2					Step 2					Step 2				
Constant	5.82(1.26)				Constant	5.66(1.05)				Constant	2.04(0.59)			
Religious practice	− 0.92(0.39)	0.39	0.18–0.87	*	Ethnicity	− 0.39(0.12)	0.67	0.53–0.85	**	Age	0.37(0.15)	1.46	1.08–1.97	*
Employment	− 0.70(0.37)	0.49	0.24–1.03	ns	Religious practice	− 0.92(0.30)	0.39	0.22–0.73	**	Ethnicity	− 0.27(0.10)	0.77	0.63–0.94	*
					Step 3					Step 3				
					Constant	6.82(1.21)				Constant	3.67(0.95)			
					Ethnicity	− 0.36(0.12)	0.69	0.55–0.88	**	Age	0.49(0.16)	1.63	1.19–2.23	**
					Religious practice	− 0.89(0.30)	0.41	0.22–0.74	**	Ethnicity	− 0.20(0.12)	0.82	0.65–1.02	ns
					Gender	− 0.76(0.37)	0.47	0.22–0.97	*	Religious practice	− 0.81(0.29)	0.44	0.25–0.78	**
					Step 4									
					Constant	6.65(1.25)								
					Ethnicity	− 0.38(0.13)	0.69	0.53–0.88	**					
					Religious practice	− 0.93(0.31)	0.39	0.21–0.73	**					
					Gender	− 0.74(0.38)	0.48	0.23–1.01	ns					
					Age	0.12(0.15)	1.12	0.83–1.52	ns					

*SE* standard error, *OR* odds ratio, *95% CI for OR* 95% confidence interval for the odds ratio, lower-upper, *ns* no statistically significant

*P < 0.05; **P  < 0.01; ***P < 0.001

**Table 5:** Binary logistic regression analysis for variables predicting giving permission to the boy/girl of the vignette (contraceptives and refusal of life-sustaining treatment) to make a decision regarding their medical treatment [B(SE), OR, 95% CI of OR, *P* value]

Contraceptives					Refusal life-sustaining treatment				
	B(SE)	OR	95% CI	*P*		B(SE)	OR	95% CI	*P*
Step 1					Step 1				
Constant	0.95(0.26)				Constant	2.03(0.33)			
Ethnicity	− 0.20(0.07)	0.82	0.72–0.93	**	Ethnicity	− 0.27(0.08)	0.77	0.65–0.89	**
Step 2					Step 2				
Constant	3.81(0.64)				Constant	3.52(0.64)			
Ethnicity	− 0.07(0.08)	0.93	0.79–1.09	ns	Ethnicity	− 0.21(0.09)	0.81	0.68–0.96	*
Religious practice	− 1.23(0.21)	0.28	0.19–0.42	***	Religious practice	− 0.64(0.19)	0.53	0.36–0.78	**
Step 3					Step 3				
Constant	6.71(1.04)				Constant	3.14(0.72)			
Ethnicity	− 0.17(0.09)	0.84	0.71–1.00	ns	Ethnicity	− 0.17(0.09)	0.84	0.70–1.00	*
Religious practice	− 1.44(0.26)	0.24	0.14–0.39	***	Religious practice	− 0.08(0.22)	0.44	0.29–0.68	***
Type of religion	− 1.84(0.42)	0.16	0.07–0.36	***	Age	0.30(0.12)	1.35	1.06− 1.73	*
Step 4					Step 4				
Constant	6.08(1.15)				Constant	7.17(1.42)			
Ethnicity	− 0.15(0.09)	0.86	0.72–1.03	ns	Ethnicity	− 0.39(0.13)	0.67	0.52–0.86	**
Religious practice	− 1.53(0.27)	0.22	0.13–0.37	***	Religious practice	− 1.27(0.33)	0.28	0.15–0.54	***
Type of religion	− 2.05(0.43)	0.13	0.05–0.29	***	Age	0.17(0.14)	1.18	0.89–1.56	ns
Education	0.49(0.15)	1.63	1.22–2.17	**	Type of religion	− 1.31(0.43)	0.27	0.11–0.63	**

*SE* standard error, *OR* odds ratio, *95% CI for OR* 95% confidence interval for the odds ratio, lower-upper, *ns* no statistically significant

*P < 0.05; **P <0.01; ***P < 0.001

There were significant associations between ethnicity and the following answers (with White British participants having the lowest rates for all of them): (a) ‘life should be prolonged^’^ as a reason to refuse permission to allow the terminally ill young person to refuse life-sustaining treatment [South Asian (8/46, 17.4%), Spanish (32/195, 16.4%), Central/South American (6/64, 9.4%), White British (3/80, 3.8%)] (*F* = 10.7, *P* < 0.05); (b) ‘I don’t agree with sexual relationships outside marriage’ as a reason to refuse permission to get contraceptives [South Asian (27/51, 52.9%), Spanish (63/194, 32.5%), Central/South American (11/65, 16.9%), White British (4/80, 5.0%)] (*F* = 47.0, *P* < 0.001); (c) ‘I don’t agree with the use of contraceptives’ [Spanish (48/194, 24.7%), Central/South American (11/65, 16.9%), South Asian (1/51, 2.0%), White British (0/80, 0%)] (*F* = 43.4, *P* < 0.001).

### Religion

Hypothesis 2 argued that more frequent religious attendance would be associated with higher ages at which young people will be regarded as able to make treatment decisions. Statistically significant age differences were found in all the vignettes. Participants without religious affiliation had the lowest mean age for all the cases, and those with frequent religious attendance had the highest mean age for most of the scenarios (Table 2). Statistically significant differences were also found regarding the percentage of letting the young person make the decision in all the vignettes with those who attended religious services frequently having the lowest percentage. Frequency of attending religious services was also found to be a statistically significant predictor for all five vignettes (Tables 4, 5).

Muslim participants gave permission statistically significantly less frequently than Christians for the refusal of life-sustaining treatment and contraceptives scenarios (Table 3). Similarly, type of religion did also reach a statistically significant level in the regression analysis for these two scenarios (Table 5).

There were also significant associations between different frequencies of attending religious services and the following responses (with participants with frequent practice having the highest rates for all of them): (a) ‘life should be prolonged’ (terminally ill scenario) [no religion (1/33, 2.0%), never (0/18, 0.0%), infrequent (6/69, 8.7%), frequent (42/259, 16.2%)] (*F* = 8.7, *P* < 0.05); (b) ‘I don’t agree with sexual relationships outside marriage^’^ (contraceptive vignette) [no religion (0/33, 0.0%), never (2/19, 10.5%), infrequent (4/71, 5.6%), frequent (97/261, 37.2%)] (*F* = 53.1, *P* < 0.001); (c) ‘I don’t agree with the use of contraceptives’ [no religion (0/33, 0.0%), never (2/19, 10.5%), infrequent (1/71, 1.4%), frequent (55/261, 21.1%)] (*F* = 28.6, *P* < 0.001).

### Education

The group of participants with university education had the lowest mean age at which adolescents were thought to be able to make medical decisions for all scenarios (hypothesis 3), reaching a statistically significant level for the operation, psychiatric admission and contraceptives vignettes (Table 2). Statistically significant differences between the groups having different levels of education were found for the latter scenario: those with a university degree had the highest percentage of answering ‘yes’ to allowing the girl to have the prescription (Table 3). This variable was also a statistically significant predictor for this vignette (Table 5).

There was a statistically significant association between the level of education and answering ‘I don’t agree with the use of contraceptives’: those with university education had the lowest rate [school (29/106, 27.4%), further education (15/87, 17.2%), university (16/194, 8.2%)] (*χ*^2^ = 19.4, 2, *P* < 0.001).

### Employment and Living Arrangements

Those participants who were employed were more likely to let the young person make the treatment decision regarding the uptake of psychotherapy sessions and receiving a contraceptive prescription than those who were unemployed (Table 3). Statistically significant differences were revealed among different living arrangements, with those who lived in local authority accommodation or were homeless having the highest level of granting permission for getting a contraceptive prescription [local authority/homeless (15/21, 71.4%); rented (64/96, 66.7%); owned/mortgaged (136/264, 51.5%)] (*χ*^2^ = 8.61, 2, *P* < 0.05). Nevertheless, these variables failed to become statistically significant predictors (Tables 4, 5).

### Gender and Age

Statistically significant gender differences were found: female participants were less likely to give permission to the young person to be admitted to a psychiatric hospital but were more likely to let the boy refuse life-sustaining treatment than their male counterparts (Table 3). Nevertheless, gender did not become a statistically significant predictor for these two vignettes (Tables 4, 5).

Statistically significant associations were found between age and letting the young people decide for themselves: the oldest group (over 65 years old) were the ones who gave more permission for the operation, hospital admission and refusal of life-sustaining treatment vignettes but the younger groups (18–30 and 31–45 years old) were the ones with the highest percentage for allowing the girl to obtain a contraceptive prescription (Table 3). Age only was also a statistically significant predictor for the operation scenario (Table 4).

No statistically significant associations were found for the following variables: civil status and having children.

## Discussion

Socio-cultural variation in adults’ attitudes to adolescents’ ability to make decisions about medical interventions was clearly found in community samples in Spain and in the UK. Our first and second hypotheses were confirmed: (1) White British had statistically significantly the lowest mean ages and the highest rate for granting permission to the young person compared with the other ethnic groups in all the scenarios; (2) participants who attended religious services frequently had statistically significantly the highest mean age in most of the scenarios, and the lowest rate for letting the young person make treatment decisions in all the vignettes. Our third hypothesis was born out by the results to a lesser extent: those with university education had statistically significantly the lowest mean age at which the young people were thought to be able to make a decision, but only for three vignettes (psychiatric admission, operation and contraceptive prescription) and they had the highest rate for granting permission for the contraceptive scenario only. It was striking that regression analysis consistently showed that the level of religious attendance was a statistically significant predictor for all five vignettes. Ethnicity predicted the granting of permission for two vignettes: for the psychiatric hospital admission and refusal of life-sustaining treatment scenarios vignettes; and education level was only a predictor for the contraceptive scenario.

### Cultural and Religious Factors

Cultural factors shape the value people place on medical treatment, influencing the extent to which the involvement of children in treatment decision-making is accepted (Blotcky et al. 1985; Chesler et al. 1986). When taking the sample as a whole, four out of the five vignettes had mean ages above 14 years (when participants thought they should be given permission to choose the treatment), and permission was refused in significant proportions (ranging from almost 10%, for the psychotherapy vignette, to almost 45% for the contraceptive one). Nevertheless, much cultural variation was found with regard to the ages and rates of giving permission (with White British and those without religious affiliation giving the lowest ages and the highest rates of granting permission).

Varying parenting styles may also contribute to these observed differences as an authoritarian style tends to oppose child autonomy, fostering compliance with parental wishes (Baumrind 1991; Danso et al. 1997), in contrast with an authoritative one which fosters children’s independence (Santrock 2007). Parenting orientations are in turn influenced by parents’ cultural and religious background, for example, Catholics’ tendency to devalue autonomy in children (Ellison and Sherkat 1993) or Muslim parents’ tendency to adopt an authoritarian style (Dwairy 2006). This is ultimately of clinical importance as research has consistently shown that an authoritative parenting style predicts good psychosocial outcomes across the studied ethnic groups (Hispanic American, European, African and Asian groups) (Steinberg et al. 1995; Steinberg et al. 1992).

Variation in the individuals’ rights to make decisions in collectivist cultures, in contrast with Western cultures, might be behind these differences and it is clear that differences exist across cultures in the UK. For example, young second-generation South Asian women in Britain experience a lack of ability to make individual decisions and lack of control, which was distressing for them. They also indicated that their parents’ understanding of adulthood was different from their White peers’ and that it was understood not in terms of age but in terms of marital and occupational status, and living arrangements (Gupta et al. 2007).

However, an alternative perspective on the findings is that although statistically significant ethnic and cultural differences have been identified, the differences in age when young people are regarded as being able to consent is relatively small. This age difference was only just over 1 year for the consent psychotherapy vignette, and just over 3 years for the consent to life-sustaining treatment. This is consistent with the view that across cultures, with increasing age and maturity, adolescents should have greater ability to make sound judgements regarding health interventions. This is also consistent with cross-cultural research with adolescents suggesting that their rights and personal expression of autonomy is widely recognized. In effect, viewing societies and cultures unidimensionally along a continuum of individualism and collectivism is too limited (Smetana 2011).

### Mental Health Interventions

The vignette regarding the boy receiving psychotherapy sessions had the highest total rate for granting permission (90.2%) as well as the lowest mean age when he would be considered mature enough to make the decision (13.34 years old). A study comparing decision-making about attending Child and Adolescent Mental Health Services (CAMHS) between 14/15-year-olds and 16-year-olds showed that there were no differences between both groups in terms of perceived ability to consent to attending CAMHS (Paul et al. 2008). There was statistically significant variation among the different ethnic and religious groups in the two mental health scenarios (psychotherapy and psychiatric hospital admission). The relatively young age when adolescents were permitted to consent to psychotherapy may reflect that this intervention was regarded as not having the potential for resulting in harm, and given its delivery over a number of sessions, could be stopped. The greater age for hospital admission might reflect that this is regarded as a more harmful intervention both in terms of stigma, but also actually risks that might be encountered once admitted.

### Contraception

Attitudes to contraception vary widely between cultures with many factors explaining this variance, such as gender relationships, views about the body and sexuality, and religious beliefs (Helman 2007). Religious practice and type of religion variables also predicted granting permission in this vignette. In some highly religious sectors of Western society, young women obtaining a contraceptive prescription without their parents’ consent have become symbols of a world that has lost its moral compass (Ehrlich 2006). The Catholic Church’s teachings strongly oppose the use of contraceptives and call for sexual abstinence before marriage. On these lines, the influence that religion exerts on attitudes towards non-sexuality (Cochran and Beeghley 1991) was highlighted by the significant associations found in our study between different levels of religious practice and some reasons behind their reluctance to grant permission (‘I don’t agree with sexual relationships outside marriage^’^ and ‘I don’t agree with use of contraceptives’), with participants with frequent practice having the highest rates of not granting permission.

### Refusal of Life-Sustaining Treatment

The complexities of the decision-making capacity of adolescents were highlighted by several public cases of children with life-threatening conditions who were refusing medical treatment (Mercurio 2007; Johnston 2009). In the competency assessments, previous experience of surgical interventions was taken into account (Johnston 2009). Similarly, the fictional character in the study’s vignette also underwent many operations and hospitalisations and this past experience might have contributed to the high overall rate of giving permission to him to refuse treatment (almost 75%).

In our study, participants with frequent attendance at religious services/ceremonies had the highest rates for arguing that the terminally ill boy’s life ‘should be prolonged’ was a reason for not letting him refuse his treatment. Interestingly, interviews with adolescents who were facing end-of-life decisions also mentioned their religious beliefs (in particular their belief in God and the certainty of life after death) as influencing their decision process (Hinds et al. 2001).

### Study Limitations

There were a number of limitations to the study. Firstly, regarding sampling, the samples were not nationally representative (they were selected as they provided wide ethnic, religious and socio-demographic variation). The Spanish sample was not nationally representative as many were recruited from Catholic colleges: the frequency for attending religious services (on weekly bases or more) was much higher in our sample (87.0%) than the national one (20.4%) and the sample’s percentage for those who never practise or who infrequently practise was much lower than the national one (11.5% and 29.0%, respectively). Nevertheless, the level of church attendance was similar to almost 40% of the Spanish population and so may be generalizable to a proportion of the population.^6^

Similarly, the study’s UK sample was more religious than the national average: a British survey showed that, in 2008, 20.2% of those who stated that they belonged to, or were brought up in a religion, attended a religious service at least once a month (excluding special occasions such as baptisms, weddings or funerals) while our sample showed that 32.1% of White British (Christian) and 66.0% of South Asians (Muslim sample obtained from a Mosque) attended services at least once a week. Nevertheless, a survey in England and Wales (2008–2009), which asked participants about their attendance at religious services and which offered data for different religious affiliation, showed much more similar data to our sample: 32% of Christian and 80% of Muslim responded affirmatively (Perfect 2011).

The study has only considered the frequency of attending religious services as a measure of the participants’ religiosity; however, this captures only a limited aspect of religiosity as the latter is a multi-dimensional construct notoriously difficult to measure, involving beliefs, practices and the religious community (Dollahite et al. 2004). Our study, like most studies do, has focused on one dimension of religiosity.

Hypothetical case vignettes were used in the survey. Although this is an established method for eliciting attitudes and beliefs, responses may not reliably describe their actual behaviour. In-depth interviews and data analysis using qualitative methods would have contributed to a more comprehensive understanding of people’s answers regarding young people’s age of consent for different health interventions and their attitudes underpinning them. Moreover, certain characteristics of the different young people portrayed in the vignettes could have added potential confounders such as their gender.

Although the participants were only asked to give the age of consent, we are mindful that age alone is not the only aspect to take into account when assessing decision-making capacity. Other aspects such as children’s social and cognitive development should be also taken into consideration (McCabe 1996).

Finally, it is accepted that the exploratory nature of the study and multiple hypotheses resulted in difficulty in sample size estimation at the planning stage. There was a risk of Type 1 (false positive findings) and Type 2 errors (false negative findings) in view of the small cell sizes for some of the analyses. Moreover, the potential interaction between the socio-demographic factors also needs to be taken into account (e.g. ethnicity and religion are likely to be correlated, as are education and employment and living arrangements).

### Clinical Implications

Our study suggests that there are social, cultural and religious variations in people’s attitudes to age of consent for health interventions. However, for youngsters who were permitted to consent to the intervention, the age of consent clustered around a few years in the adolescent phase of development. In clinical contexts there may be greater variation than is established here. This is especially important as the cultural diversity of families seen in the metropolitan cities of the UK and many other countries is far greater than that investigated here. This means there may be clinical encounters in which parents will not consent to a recommended intervention in mental or general health settings. The adolescents themselves may wish to accept the intervention, and this may generate conflict between parents and clinicians. Alternatively, adolescents may refuse medically recommend interventions for life-threatening conditions. This may also generate conflict between the adolescent on the one hand and carers and medical staff on the other. In this situation, the child and adolescent mental health paediatric liaison team may be involved to evaluate the mental health of the adolescent, to exclude, for example, a depressive disorder that results in suicidal thinking. In addition, the liaison team may also be asked to comment on the capacity of the youngster, to establish whether they do have sufficient understanding and cognitive abilities to give or withhold consent. The cultural context for such difference of views requires skilled exploration and management of adolescents and parents views, within an understanding of child rights and legal aspects of consent. Additional professionals and respected individuals, such as priests, from the communities or wider family may be needed to help resolve divergent views.

## Footnotes

1 Authoritarian parents are obedience- and status-oriented, and have the expectation to have their orders followed (Baumrind, 1991) while authoritative parenting is more child-centred, fostering children’s independence while still keeping up boundaries (Santrock, 2007).

2 We are aware that comparing different cultures in terms of individualism-collectivism has been seriously questioned (e.g. McSweeney, 2002). Its use is a simplification for the study purposes and we are mindful that both individualistic and collectivistic orientations can co-exist in the same culture (Kim et al., 1994).

3 Valencia is the third largest city in Spain. About 1,175,000 people live in the Valencia urban area (Demographia, 2011) and 2,300,000 in the Valencia metropolitan area (Organization for Economic Cooperation and Development 2006).

4 When the ANOVA homogeneity of variance of assumption was broken (Levene’s test was significant), Welch’s *F* correction was used: the Welch test is robust when homogeneity of variance has been violated (see for example Tomarken & Serlin 1986; Field 2009).

5 ‘Leave to remain’ is the standard legal term for describing migrants who have acquired legal rights to settlement in a country, typically when they come from a country with which the host nation does not have a general agreement for migration.

6 A survey undertaken in Spain, amongst those with Spanish nationality, showed that 76.0% declared themselves as ‘Catholic’, 13.0% as ‘non-believers’, 7.3% as ‘atheists’ and 2.1% as ‘believers of other religions’ (Centre for Sociological Investigations, 2009b). When those who defined themselves as Catholic or believers of other religion were inquired about the frequency with which they went to mass or other religious services without counting those occasions related to ceremonies of a social kind such as wedding, communions, funerals, etc. Half answered that ‘almost never’ (50.0%), ‘almost every Sunday and festivities’ (18.0%), ‘several times a year’ (17.7%), ‘at least one a month’ (11.3%) and ‘several times a week’ (2.4%) (Centre for Sociological Investigations, 2009a).
